# Association of Overweight and Obesity with Impaired Executive Functioning in Mexican Adolescents: The Importance of Inhibitory Control

**DOI:** 10.3390/healthcare12141368

**Published:** 2024-07-09

**Authors:** Yatzeny Guadalupe Ruiz-Molina, Josué Herrera-Ávila, Josué Vidal Espinosa-Juárez, Héctor Armando Esquinca-Avilés, Juan Gabriel Tejas-Juárez, Elena Flores-Guillén, Luis Alberto Morales-Martínez, Alfredo Briones-Aranda, Betsabé Jiménez-Ceballos, José Alfredo Sierra-Ramírez, Refugio Cruz-Trujillo

**Affiliations:** 1 Sección de Estudios de Posgrado e Investigación, Escuela Superior de Medicina, Instituto Politécnico Nacional, Ciudad de Mexico 11340, Mexico; molina_1895@hotmail.com (Y.G.R.-M.); josueheav05@gmail.com (J.H.-Á.); 2 Escuela de Ciencias Químicas, Universidad Autónoma de Chiapas (UNACH), Carretera Panamericana Ocozocoautla-Cintalapa Km. 2.5, Ocozocoautla de Espinosa 29140, Mexico; josue.espinosa@unach.mx (J.V.E.-J.); hesquinc@unach.mx (H.A.E.-A.); 3 División Académica Multidisciplinaria de Comalcalco, Universidad Juárez Autónoma de Tabasco, Tabasco 86658, Mexico; juan.tejas@ujat.mx; 4 Facultad de Ciencias de la Nutrición y Alimentos, Universidad de Ciencias y Artes de Chiapas (UNICACH), Libramiento Norte-Poniente 1150, Col. Lajas Maciel, Tuxtla Gutiérrez 29039, Mexico; elena.flores@unicach.mx (E.F.-G.); luis.morales@unicach.mx (L.A.M.-M.); 5 Facultad de Medicina Humana Campus II, Universidad Autónoma de Chiapas (UNACH), Décima Sur esquina Calle Central S/N, Tuxtla Gutiérrez 29050, Mexico; alfredo.briones@unach.mx; 6 Clínica de Trastornos del Sueño, Universidad Autónoma Metropolitana, Unidad Iztapalapa (UAM-I), Av. San Rafael Atlixco 185, Col. Leyes de Reforma, Iztapalapa, Ciudad de Mexico 09340, Mexico; betsabe.jmz@xanum.uam.mx; 7 Departamento de Químicos Farmacobiólogos, Universidad Pablo Guardado Chávez (UPGCH), Libramiento Norte Oriente No. 3450, Tuxtla Gutiérrez 29040, Mexico

**Keywords:** adolescence, executive function, inhibitory control, obesity, overweight, orbitomedial cortex, physical activity

## Abstract

Overweight and obesity are major public health issues worldwide, including in Mexico, particularly among adolescents. This study aimed to analyze the associations between nutritional status and impaired executive function (EF) in Mexican adolescents. A case–control study was conducted with 98 male and female adolescents, categorized into normal weight and overweight/obese groups based on body mass index. EF was assessed using the BANFE-2 test. The prevalence of overweight and obesity was 54.3%. The EF assessment revealed that 82.45% of the overweight/obese group exhibited mild-to-severe impairment, compared to only 36.58% in the normal weight group (X^2^ = 21.69, *p* < 0.0001). In the inhibitory control assessment, adolescents with overweight and obesity performed worse than their normal-weight counterparts. Specifically, females with overweight/obesity scored lower than females with normal weight on the risk–benefit processing test. The risk of severe EF impairment significantly increased with the presence of overweight/obesity (OR = 7.8, *p* < 0.0001). These findings indicate that EF, particularly inhibitory control and risk–benefit processing, is impaired in adolescents with overweight or obesity.

## 1. Introduction

The increasing prevalence of overweight and obesity represents a significant global public health issue. According to the World Health Organization, these conditions involve the atypical or excessive buildup of adipose tissue, which can have detrimental effects on health [1]. The rising rate of obesity and overweight among adolescents underscores the urgency of addressing this problem. Globally, there is a staggering 340 million adolescents that suffer from obesity [2]. In Mexico, this issue is also prevalent, as evidenced by statistics obtained from the National Health and Nutrition Survey (ENSANUT) 2021, which reported a prevalence rate of 24.7% for overweight and 18.2% for obesity among adolescents within this population group [3]. Specifically, in Chiapas state, the prevalence rates are 22.1% for overweight and 7.6% for obesity among adolescents, based on their body mass index (BMI) [4].

Anthropometric measures are crucial for assessing the nutritional well-being of a population. The most commonly used anthropometric measurement in adolescents is BMI, which serves as an indirect indicator of body fat and provides valuable insights for identifying overweight and obesity. The categorization of overweight and obesity based on BMI varies across different age groups and genders, including newborns, children, and adolescents [5]. Beyond the physical implications, a notable connection between obesity and executive function (EF) has emerged, establishing itself as an important area of research [6].

Excess weight has repercussions on various systems, and these effects can manifest even at an early age. During adolescence, it can impact brain and emotional development [1]. It is important to address this issue to promote health and well-being at all ages. For example, adolescents who are overweight or obese may experience negative effects on brain structure, such as an increase in the size of the amygdala and accumbens [7]. Additionally, the prefrontal cortex (PFC), specifically the orbitofrontal cortex (OFC), may have reduced volumes [8,9]. Consequently, it is unsurprising that adolescents with obesity often face psychological issues, including depression, anxiety, decreased self-worth, impaired decision-making, dissatisfaction with body image, and eating disorders [10].

Cognitive processes of self-regulation, including monitoring, thought control, and goal-directed activities, are encompassed under EF [11]. Three essential domains of EF have been identified: inhibitory control, working memory, and cognitive flexibility. These domains form the basis for the development of higher-order cognitive processes, such as planning, reasoning, decision-making, selective attention, and impulse control [12,13].

Research on EF capabilities has been conducted in both adults and adolescents. Notably, some EF processes continue to develop during adolescence as young individuals encounter new problems and responsibilities [14]. A deficiency in EF has been proposed as a significant risk factor for the development and perpetuation of obesity. Particularly, self-regulation, which involves inhibitory control over impulsive food intake, EF emerges as a critical component in the regulation of body weight [11,15]. Poor eating behaviors and a lower commitment to an active lifestyle have been shown to be associated with challenges in exercising inhibitory control [16]. These findings highlight the importance of including EF as a crucial aspect in understanding the risk factors associated with obesity and overweight, thereby opening new perspectives for interventions aimed at strengthening these cognitive abilities.

Four systematic reviews of EF impairments in adolescents with obesity and overweight have indicated that those with these conditions show poor performance in working memory, inhibitory control, planning, attention, mental flexibility, and reward sensitivity [11,12,17,18]. For instance, adolescents with overweight have been shown to perform worse on EF assessments compared to their normal-weight counterparts, exhibiting difficulties in areas such as choice reaction time, sustaining attention, and inhibiting impulsive responses [19].

Notably, BMI is inversely related to EF performance [20]. An evaluation of inhibition of go/no-go responses to appetitive food stimuli among adolescent females found that individuals with a higher BMI responded significantly faster to food consumption cues and made errors when their responses to dessert pictures were not inhibited [21]. Another study of adolescents with overweight revealed that as BMI increased, there was a decrease in card game switching compared to their normal weight counterparts [22]. These findings suggest that being overweight may have a detrimental effect on the development and functioning of EF in adolescents.

Despite the considerable variability in assessment methods, the studies collectively demonstrate an association between higher childhood BMIs for age and poorer performance on inhibitory control tasks [23,24,25,26,27,28]. Nevertheless, it is crucial to recognize that several investigations conducted on adolescents with overweight and obesity have been unable to establish any associations with impaired EF [29].

Despite the existence of several studies that have provided insights into the associations between obesity and EF in adolescents, the comprehension of this influence continues to face obstacles and requires further investigation. Given the factors mentioned above, this study seeks to evaluate the association between nutritional status and impaired EF in Mexican adolescents, emphasizing the importance of inhibitory control in this interaction. This will enhance understanding of the intricate association, offering a perspective that will aid in the formulation of effective strategies to promote comprehensive health and well-being at this critical phase of human development.

## 2. Materials and Methods

### 2.1. Participants

The individuals included in this research were selected from two public high schools in Ocozocoautla de Espinosa, Chiapas, Mexico. At the outset of the study, participants received a detailed explanation of the project’s objectives. Two documents were issued: a letter of assent for the adolescents and a letter of informed consent for the parents. Both documents were duly signed and returned.

The sample size was obtained using the following formula: n = [Z^2^ × Npq]/[e^2^(N − 1) + Z^2^pq]. N = the population size (840 students), Z^2^ = confidence level (95%), p = probability in favor (50%), q = probability against (50%), e = standard error (5%). Based on these parameters, the calculated sample size was 266 adolescents. However, 168 adolescents who did not complete the anthropometric measurements, physical activity questionnaire, and BANFE-2 tests were excluded. A total of 98 students voluntarily participated in the study and completed all the tests. The study adhered strictly to the principles outlined in the Declaration of Helsinki. Additionally, the protocol received approval from the Ethics Committee of the Escuela Superior de Medicina del Instituto Politécnico Nacional (ESM-CEI-01/26-03-22 Version 2.0).

### 2.2. Evaluation Instruments

#### 2.2.1. Body Mass Index (BMI)

Anthropometric measurements were conducted by university students from UNICACH who were in the final year of their Bachelor’s program in nutrition. Prior to this, they underwent a standardization course covering anthropometric techniques, which was conducted and supervised by appropriately trained personnel. The participants’ weights and heights were measured, and their BMIs were calculated by dividing weight (kg) by the square of height (m^2^). For the categorization of nutritional status, we use the cut-off points of the BMI for age Z-scores or standard deviations (SD) established by the 2007 WHO for adolescents: normal weight (<−2 to ≤+1 SD) and overweight or obesity (>+1 SD) [30].

#### 2.2.2. BANFE-2

The study involved administering a neuropsychological assessment called the Battery of Frontal and Executive Functions 2 (BANFE-2) to the adolescent participants [31]. Recently published studies have utilized either the BANFE-1 or BANFE-2 test to analyze EF [13,32,33,34,35]. This battery has been validated for the Mexican population, considering age and education level across a wide age range spanning from 6 to 80 years. The inter-rater reliability among applicators is 0.80. The battery comprises fifteen assessments related to EF, categorized into three frontal regions: orbitomedial (mOFC), anterior prefrontal, and dorsolateral. In this study, we specifically focus on mOFC by analyzing inhibitory control, rule-following, and risk–benefit processing. The BANFE-2 test classifies EF into four categories based on cutoff points of the normalized total score: 116 and above (high normal), 85–115 (normal), 70–84 (mild–moderate impairment), and 69 or below (severe impairment) [31].

##### Stroop form A and B

These tests assess inhibitory control skills, evaluating the adolescents’ ability to manage their impulses. Two tests were conducted to evaluate this inhibitory control: (1) Stroop A, a test assessing participants’ capacity to inhibit automatic responses. During the test, participants were given a sheet with six words representing color names. They were instructed to read each word in the column except for the highlighted word. Instead, participants were required to state the color in which the phrase was printed rather than the written word. (2) In Stroop B, participants were required to read aloud the columns of words as instructed by the evaluator. However, they were directed to modify their answer when the word “color” was uttered. In this scenario, participants were expected to identify the print color rather than the written word. The time of execution, hits, and errors were documented [13,31].

##### Card Game

This assessment focuses on the ability to recognize and avoid hazardous choices, as well as to recognize and sustain advantageous choices. During this task, participants were instructed to maximize their score by selecting cards with values ranging from one to five. The examination involves making iterative judgments throughout a series of 10 blocks, consisting of 5 blocks that create points and 5 blocks that deduct points. Participants developed their own judgment in selecting each card, meticulously evaluating the corresponding risks and advantages, with the objective of attaining the highest score possible. The calculation included determining the percentage of risk cards, as well as the total score achieved during the execution of the card game assignment [31,32].

##### Maze Test and Card Sorting

These assessments evaluate an individual’s capacity to adhere to boundaries and comply with prescribed rules. The maze test consists of five labyrinths that exhibit varying degrees of difficulty. Participants were instructed to complete these labyrinths in the shortest feasible time while avoiding contact with or crossing the walls. This task was designed to assess their ability to regulate impulsivity. The frequency of contact between the pencil line and the walls of the labyrinth was documented.

Regarding the card sorting test, a set of four cards was provided, each featuring distinct geometric shapes (circle, cross, star, and triangle). Each card was distinguished by two attributes: numerical value and color. Participants were provided with a collection of 64 cards that had identical features. They were then directed to organize these cards according to one of four base cards that were shown on a page. The criteria for arranging the cards were determined by the participant, which may be based on color, shape, or number. In the absence of any perceptual pattern to influence decision-making, each card exhibited an equal probability of being associated with all three criteria. Participants organized the 64 cards based on their perception of the relationship or ranking with the corresponding sheets. Maintenance errors were documented when the correct sequence was not maintained, and a decision was made to modify the ranking criterion after at least three consecutive hits [31,33].

#### 2.2.3. Physical Activity

The ENSANUT 2019-validated physical activity questionnaire was administered to adolescents and adults (aged 15 to 69 years) in the Mexican population [36]. The questionnaire assessed the amount of time spent performing activities that require moderate physical effort, as well as the duration of walking over the last seven days, with each session lasting a minimum of 10 consecutive minutes. Individuals who engaged in a minimum of 60 min of physical activity at a moderate-to-vigorous level on all seven days of the week, totaling 420 min, were categorized as active [37,38].

### 2.3. Procedures

The participants in the research were instructed to wear lightweight clothing and observe a fasting period before the measurements. Furthermore, they were asked to provide their name and age. The procedures were conducted from 8 to 11 a.m. in designated spaces within the schools to ensure privacy. Researchers collected weight and height measurements from the subjects while they were in an upright posture, without shoes, and after a controlled exhale.

The weight of the adolescents was measured using a calibrated scale (Tanita BC-601) with a precision of ±100 g. Height was measured in centimeters using a stadiometer (Seca, Hamburg, Germany) with a precision of ±1 mm. Participants were instructed to stand upright, ensuring contact between their heels, buttocks, back, and occipital area with the vertical plane of the stadiometer. During the assessment, participants took in a deep inhalation to minimize the compression of intervertebral disks, considering the Frankfurt plane to enhance measurement precision.

The BANFE-2 battery was administered to adolescent participants [31]. The exclusion criteria included head trauma, psychiatric disorders, and the use of drugs affecting the central nervous system (CNS).

The BANFE-2 examination was conducted individually by examiners who had received prior training. The test took place in a quiet, spacious, and well-illuminated environment within the designated facilities. The average duration for each student to complete the exam was 20 min.

The implementation of the BANFE-2 batteries involved utilizing various resources, including an instruction manual, a recording procedure, application sheets, a set of cards, cards for card sorting, sheets containing labyrinth, and a stopwatch. The examination commenced with administering a set of inquiries aimed at gathering personal and medical data from the subjects, as per the established procedure. Subsequently, the exams were conducted, starting with the completion of the labyrinth and then proceeding to the card-sorting assignment. Following the utilization of the Stroop effect sheet form A, the subsequent activity involved the “Lowa” card game. Finally, the Stroop effect sheet form B was implemented. The students successfully completed the physical activity questionnaire during the preceding session.

### 2.4. Statistical Analysis

The statistical analysis was performed using GraphPad Prism (GraphPad Software version 8.0, LLC, Boston, MA, USA) and IBM SPSS Statistics Software version 28.0 (IBM Corporation, New York, NY, USA). The normality of the variables was assessed using the Kolmogorov–Smirnov test. Descriptive statistics such as percentages, means, and medians, along with their corresponding standard deviations and interquartile ranges (IQRs), were used to represent the variables. The variables age, height, weight, and BMI were analyzed using Student’s *t*-test. The medians of the natural and normalized scores in the Stroop A and B subdomains, maze test, card game, and card sorting of the BANFE-2 test were compared between the normal-weight and overweight/obese groups using the Mann–Whitney U test. The X^2^ test was utilized to assess the relationship between normal weight and overweight/obesity, physical activity (active and sedentary), and EF categorization (high normal, normal, mild/moderate impairment, and severe). The calculation of risk assessment was conducted using the Odds Ratio (OR). To analyze EF, the variables were stratified by sex, considering the sex difference in anthropometric parameters and physical activity. Statistical significance was assumed at *p* < 0.05.

## 3. Results

### 3.1. Descriptive Analysis of the Population by Gender

The final sample comprised 98 adolescents, with 69 females and 29 males, who underwent anthropometric measurements, the four BANFE-2 tests, and the physical activity questionnaire. Table 1 summarizes these data. The majority of the sample was composed of females, accounting for 70.40% of the total. No statistically significant differences were found between males and females regarding age, BMI, and the prevalence of overweight/obesity (*p* > 0.05). However, noticeable disparities were observed in height, weight, and level of physical activity between genders. Specifically, males exhibited greater height, weight, and physical activity compared to females (*t* _(96)_ = 6.464, *p* < 0.0001; *t* _(96)_ = 2.674, *p* = 0.0088; X^2^ = 8.489, *p* = 0.0036, respectively). The overall prevalence of overweight/obesity was 54.3%, with a similar prevalence observed in females (63.77%) and males (44.83%). The BANFE-2 test results revealed no statistically significant differences in EF performance between males and females.

### 3.2. Overweight/Obese Adolescents Performed Worse on Executive Functioning

Table 2 presents a comprehensive overview of the anthropometric attributes, physical activity levels, and BANFE-2 assessment performance among adolescents categorized as normal weight and overweight/obese. The age of participants ranged between 16 and 17 years (mean = 16.64, SD = 0.062). No significant differences were observed in age, height, and physical activity level between the experimental groups (*p* > 0.05). Interestingly, 63.26% of the sample demonstrated insufficient physical activity.

In terms of anthropometric indicators, the overweight/obese groups exhibited significantly greater weight and BMI compared to the normal weight group (*t*_(96)_ = 12.49, *p* < 0.0001). Furthermore, 82.42% of the overweight/obese group displayed mild to moderate/severe EF impairment on the BANFE-2 test, whereas only 36.58% of the normal-weight group exhibited similar impairments (X^2^ = 21.69, *p* < 0.0001). These findings highlight the association between body composition and cognitive performance among the participants.

### 3.3. Adolescents with Overweight/Obesity had Deficits in Inhibitory Control and Decision-Making

To identify the specific areas of EF compromised in adolescents with overweight and obesity, Table 3 provides median natural and coded total scores, card sorting, Stroop A and B, a maze test, and card games. In the mOFC domain, both the natural and coded total scores of adolescents with overweight/obese were significantly lower than those of adolescents with normal weight (U = 568 and U = 566, *p* < 0.0001, respectively). Significant differences in Stroop A errors (U = 851, *p* = 0.0186) and Stroop B errors, time, and hits (U = 823, *p* = 0.0102; U = 858.5, *p* = 0.0252; U = 888, *p* < 0.0388, respectively) were identified when comparing the outcomes of the Stroop A and B test in this study group to those of normal weight adolescents.

A significant decrease in total scores on the card game test was observed among adolescents with overweight/obese compared to those in the normal weight group (U = 890, *p* = 0.0446). However, the presence of overweight and obesity did not yield any statistically significant differences in the maze test and card sorting tests. These results underscore specific changes in particular dimensions of EF among adolescents who are overweight and obese, emphasizing the need for targeted approaches in the assessment and treatment of such conditions.

### 3.4. Women with Overweight/Obesity Exhibited Deficits in Inhibitory Control and Decision-Making, Whereas Men with Overweight/Obesity Displayed Deficits Only in Inhibitory Control

A comprehensive examination of EF in adolescents, categorized by gender and BMI, is shown in Table 4. Statistically significant differences are observed between males and females with normal weight and overweight/obesity on several measures. A comparison was made between adolescents with overweight/obesity and their normal-weight counterparts in the mOFC domain. Specifically, males [183 (177.5–190) vs. 192 (191–194.8), U = 35.50, *p* = 0.0018] and females [185 (177.3–190) vs. 190 (184–195), U = 324, *p* = 0.0042] exhibited significantly reduced natural total scores.

Similarly, the encoded total score of adolescents with overweight and obesity was significantly lower in both genders than the normal weight group [U = 323.5, *p* = 0.0041; females: 69 (52.5–83) vs. 83 (66–98); males: 63 (46.5–83) vs. 89 (86–97.25); U = 35.50, *p* = 0.0018]. Moreover, a notable difference in errors [1 (0–2.75) vs. 3 (1–6.5), U = 55.50, *p* = 0.0282] and hits [80 (79–82.75) vs. 77 (75–81.5), U = 49, *p* = 0.0143] were observed in Stroop A between males with normal weight and overweight/obesity. In Stroop B, a significant difference in time dedicated by men with overweight/obesity and their normal-weight peers was observed [94 (77.5–104) vs. 73.5 (68.5–82.75), U = 54.50, *p* = 0.0291]. Additionally, women with normal weight committed fewer errors than peers with overweight/obesity [1 (0–2) vs. 2 (0.25–4), U = 378.5, *p* = 0.0280]. Regarding the card game, females with overweight and obesity had substantially lower total scores than females with normal weight [23 (16.25–29) vs. 28 (21–42), U = 388.5, *p* = 0.0433]. The findings emphasize the influence of weight status and gender on EF in adolescents, highlighting the significance of taking both aspects into account when evaluating EF in this group.

### 3.5. Adolescents with Overweight/Obesity Are at Very High Risk of Severe and Mild/Moderate Impairment in Executive Functioning

Table 5 provides a summary of the OR describing associations between body composition (normal weight and overweight/obese) and categorization EF (normal, impaired, mild/moderate, and severe impairment). The study revealed that overweight/obesity among adolescents was associated with a 7.2-fold increase in the probability of poor EF results (OR = 7.2, *p* < 0.0001). Additionally, the presence of overweight/obesity substantially raised the probability of experiencing mild/moderate impairment in EF by 6.3 (OR = 6.3, *p* = 0.002). Furthermore, the existence of overweight/obesity resulted in a substantial 7.8-fold increase in the probability of encountering severe changes in EF (OR = 7.8, *p* < 0.0001), highlighting a robust association between these factors.

## 4. Discussion

This study evaluated EF in adolescents from southeast Mexico using the BANFE-2 neuropsychological battery, taking into account their BMI. The findings indicated that 82.45% of adolescents who were overweight or obese had impairments in EF, ranging from mild-to-moderate to severe, in comparison to 36.5% of those with a normal weight. Furthermore, these results revealed that females who were overweight or obese demonstrated alterations in inhibitory control and decision-making abilities, while males displayed deficits in inhibitory control.

The assessed adolescent population exhibits a notable prevalence of overweight and obesity, with a rate of 54.3%, surpassing the most recent data documented in Mexico, which revealed a prevalence rate of 42.9% published by ENSANUT 2021 [3]. Furthermore, it exceeds the findings of research conducted among adolescents in Chiapas, Mexico, which recorded a prevalence rate of 38% for overweight/obesity [4]. It is essential to consider that the recruitment of research participants occurred after a time characterized by governmental limitations imposed due to the COVID-19 pandemic. These initiatives resulted in extraordinary circumstances that caused significant disruptions to individuals’ everyday routines, thereby increasing the prevalence of noncommunicable illnesses, such as obesity. The observed phenomena may be ascribed to alterations in daily schedules, decreased levels of physical activity, heightened sedentary behaviors, and shifts in dietary patterns resulting in the excessive intake of high-calorie foods. Consequently, these factors contribute to an elevated susceptibility to overweight and obesity [39,40,41]. In contrast, the psychological consequences of social isolation and house confinement, implemented as measures to mitigate the transmission of the virus, have been shown to elevate the probability of mental health decline. This is evident in several manifestations, such as depression, anxiety, stress, sleep disturbances, and weight gain [39,42].

The absence of physical exercise has emerged as the primary factor contributing to several chronic noncommunicable disorders, such as obesity [43]. This study reveals differences in physical activity levels between genders, consistent with other studies conducted on adolescents [44,45,46]. Observed variations in physical activity across genders partially account for the elevated occurrence of overweight and obesity in females relative to males. Importantly, no significant association was found between gender and EF. These results indicate that, within this group, gender does not appear to play a decisive role in executive abilities, which aligns with previous research [32].

We found that 82.45% of adolescents with overweight and obesity in our study exhibited EF deficiencies, varying in severity from mild/moderate to severe. Research conducted on a sample of adolescents with obesity exhibiting indications of food addiction yielded comparable findings, indicating that 73.66% of participants had moderate-to-severe deficits in EF [47]. The probability of experiencing mild/moderate and severe poor performance in EF was significantly higher in the overweight and obese groups compared to their normal-weight counterparts (OR = 7.2).

In this study, we utilized the BANFE-2 to investigate the effects of mOFC-controlled EF. Our findings indicate that adolescents with overweight and obesity exhibited notably worse performance on both natural and normalized mOFC subdomain scores compared to their normal-weight counterparts. Previous research has suggested that the CNS is not fully developed during adolescence [48]. Additionally, structural changes in brain regions such as the hippocampus, cerebellum, amygdala, and PFC have been observed in individuals who are overweight and obese [9,20,49], which may increase the likelihood of developing EF deficits. The PFC is responsible for regulating EF associated with impulse control. This includes the OFC, which plays a vital role in decision-making and the selection of actions connected to eating and rewards [50]. The mOFC subdivision within the OFC serves as a focal point for the convergence of many external inputs, including visual, sensory, and olfactory stimuli, as well as internal stimuli, such as visceral, taste, and somatosensory stimuli. The impact on this region may manifest as changes in personality and impairments in attention, learning, and working memory [51].

Furthermore, it has been demonstrated that the mOFC sends axonal projections to the nucleus accumbens and basolateral amygdala [52,53]. These projections play a crucial role in regulating the effort circuit and facilitating the process of selecting or rejecting inappropriate meals, as well as making nutritional decisions [54]. Nevertheless, there is evidence suggesting that a high BMI is associated with reduced connectivity between the ventral striatum and the OFC, which are key components of the reward system [55]. This reduced connectivity may lead to a loss in impulse control by the PFC when it comes to consuming meals that are rich in fat and sugar. This observation is supported by research conducted on women who are fasting and obese, where heightened activity was observed in the OFC, nucleus accumbens, anterior cingulate cortex, and medial prefrontal cortex when exposed to visual stimuli depicting enticing food items [56]. Consuming calorie-rich meals has been shown to stimulate brain reward areas, resulting in a pleasure sensation that serves to alleviate stress or unpleasant emotions. Moreover, the suppressive effects of both acute and chronic stress on the PFC, the cerebral region responsible for regulating behavior [42], strengthen the reciprocal association between nutritional status and EF.

Upon analysis of the various subdomains of EF, it becomes evident that inhibitory control shows a decline, as evidenced by notable modifications in Stroop A and B tests, thus supporting prior research [57]. The decline in inhibitory control is associated with an increase in food consumption and, consequently, a higher BMI in adolescents [21,58,59]. Moreover, it is conceivable that poor inhibitory control in individuals with obesity is linked to a propensity for impulsive eating [60]. The executive system in the brain is undeniably pivotal in regulating eating behavior since it is responsible for the conscious and intentional act of consuming food. The implementation of this system is crucial in limiting lifestyle behaviors that may contribute to the progress of obesity [61]. The coexistence of executive dysfunction and impaired impulse control has been identified as a significant determinant of obesity, increased food consumption, and inadequate dietary regulation [57,62,63,64]. Research conducted on individuals exhibiting dietary restriction revealed that, despite their efforts to regulate their food consumption, those with impulsive tendencies are more prone to engaging in overeating when confronted with temptation [65]. Another study demonstrated that individuals who are obese and exhibit high impulsivity were able to identify hypercaloric foods at a significantly faster rate compared to those who have a healthy weight [66]. This finding suggests that cognitive processes associated with eating control and the subsequent progression of overweight may partially bypass negative feedback control in individuals who are prone to engaging in risky eating behaviors within a society that is heavily influenced by hypercaloric foods [61,67]. Nevertheless, more investigation is needed to understand the fundamental processes that underlie these connections and their possible ramifications for cognitive well-being in the long term.

Limited research has been conducted to compare differences in EF subdomains between males and females with normal weight and overweight/obesity [32]. In the present study, the sex-stratified analysis revealed significant differences in inhibitory control and EF decision-making between normal-weight and overweight/obese females. Conversely, differences in inhibitory control were only observed between males with normal weight and overweight/obesity. These data suggest that the relationship between body composition and EF in adolescents may be influenced by gender. A comprehensive understanding of these gender differences is crucial for the development of tailored approaches that effectively address the specific needs of each demographic group. Additionally, considering the findings of a specific study that found a particularly strong association in females, it is possible that females may be more vulnerable than males to the possible adverse effects of visceral fat on cognitive function [68].

While this study presents new and interesting results, it is crucial to acknowledge many significant constraints. Initially, the study did not include additional variables that have been identified as potential factors influencing EF impairment in adolescents with overweight and obesity. The variables include sociodemographic characteristics, dietary habits, food dependency, levels of anxiety, stress, melancholy, diminished self-worth, dissatisfaction with body image, eating disorders, genetic predisposition, intestinal microbiome, post-traumatic stress disorder, and prenatal exposure to alcohol, which, although they were not considered in this research, should be further investigated in future studies involving the Mexican population [10,69,70,71,72]. Furthermore, the present investigation used BMI as an anthropometric tool, which lacks the ability to directly quantify body fat and may not effectively capture or distinguish changes in body composition, such as variations in body fat and muscle mass [17]. It is essential to consider other anthropometric measures that quantitatively assess body fat, such as waist circumference and the proportion of visceral fat as determined by bioimpedance. Additionally, the sample exhibited a higher proportion of females compared to males, thereby limiting the applicability of the findings to the broader community. It is important to mention that this research only examined the evaluation of the mOFC without including the anterior prefrontal and dorsolateral cortex. It would be interesting to consider the inclusion of these areas in future studies to obtain a better understanding of the reciprocal association between body composition and EF.

Future research should prioritize the identification of efficacious interventions for addressing adolescents with overweight and obesity, as this condition has been linked to a greater susceptibility to comorbidities previously observed in the adult demographic, including type 2 diabetes, hypertension, nonalcoholic fatty liver disease, obstructive sleep apnea, and dyslipidemia [10]. Recent research indicates that the incorporation of consistent physical activity initiatives has the potential to enhance inhibitory control and cognitive flexibility, therefore fostering compliance with a nutritious dietary regimen [73]. Similarly, research has shown adolescents with overweight who engage in weight loss programs showed enhancements in cognitive development, anthropometric measurements, anxiety, and depression [32]. Additionally, regular sports participation among young individuals is linked to improved EF in comparison to their less physically active counterparts [19]. Hence, it is essential to advocate for the adoption of healthy behaviors starting at a young age, including a well-rounded diet, consistent engagement in physical exercise, restriction of sedentary pursuits like electronic device use, and routine evaluation of anthropometric and EF measures in adolescents.

## 5. Conclusions

This research presents compelling evidence about the complex relationships between BMI and EF in adolescents, emphasizing the potential negative impact of overweight and obesity on several elements, including inhibitory control and the propensity to engage in risky behaviors. These interesting associations underline the importance of preventing overweight and obesity during adolescence. However, further studies are needed to deepen our understanding of the underlying mechanisms and to develop more effective therapies. Acquiring this information is crucial to promoting the overall well-being of adolescents and minimizing the lasting physical and mental health repercussions.

## Figures and Tables

**Table 1:** Baseline characteristics of participants.

Variables	Female (*n* = 69)	Male (*n* = 29)	*p*-Value
Age (years)	16.64 ± 0.64	16.64 ± 0.54	0.9960
Height (cm)	153.1 ± 10.93	167.0 ± 5.64	**<0.0001**
Weight (kg)	61.17 ± 10.11	68.27 ± 15.69	**0.0088**
BMI (kg/m^2^)	25.89 ± 3.65	24.31 ± 4.54	0.0717
Normal weight, *n* (%)	25 (36.23)	16 (55.17)	0.0827
Overweight/Obesity *n* (%)	44 (63.77)	13 (44.83)	
**Physical activity, *n* (%)**			**0.0036**
Sedentary	50 (72.46)	12 (41.38)	
Active	19 (27.54)	17 (58.62)	
**BANFE-2 performance grading, *n* (%)**			0.0957
Severe (≤69)	31 (44.93)	8 (27.59)	
Mild-to-Moderate (70–84)	17 (24.64)	5 (17.24)	
Normal (85–115)	20 (28.99)	14 (48.28)	
Superior (≥116)	1 (1.45)	2 (6.90)	

Abbreviations: BMI, body mass index; BANFE-2, Neuropsychological Battery of Executive Functions and Frontal Lobes. Values are expressed as mean ± standard deviation (SD). BMI categories were defined according to the WHO 2007 criteria [30]. The percentages of normal weight and overweight/obesity, physical activity, and EF were analyzed using the chi-square test. Statistically significant *p*-values are shown in bold.

**Table 2:** Anthropometrics, body composition, physical activity, and executive function of participants.

Variables	NW (*n* = 41)	OW/OB (*n* = 57)	*p*-Value
Age (years)	16.76 ± 0.55	16.56 ± 0.64	0.1098
Height (cm)	159.1 ± 7.23	155.9 ± 13.76	0.1821
Weight (kg)	55.15 ± 6.65	69.11 ± 12.29	**<0.0001**
BMI (kg/m^2^)	21.75 ± 1.72	28.07 ± 2.88	**<0.0001**
**Physical activity, *n* (%)**			0.6901
Sedentary	25 (60.98)	37 (64.91)	
Active	16 (39.02)	20 (35.09)	
**BANFE-2 performance grading, *n* (%)**			**<0.0001**
Severe (≤69)	9 (21.95)	30 (52.63)	
Mild-to-Moderate (70–84)	6 (14.63)	17 (29.82)	
Normal (85–115)	24 (58.54)	9 (15.79)	
Superior (≥116)	2 (4.88)	1 (1.75)	

Abbreviations: NW, normal weight; OW, overweight; OB, obesity; BMI, body mass index; BANFE-2, Neuropsychological Battery of Executive Functions and Frontal Lobes. Values are expressed as mean ± standard deviation (SD). BMI categories were defined according to the WHO 2007 criteria [30]. The percentages of physical activity and EF were analyzed using the chi-square test. Statistically significant results are shown in bold.

**Table 3:** Executive function performance in adolescents with normal weight and overweight/obese.

BANFE-2	NW (*n* = 41)	OW/OB (*n* = 57)	*p*-Value
**mOFC, Total Score, median (IQR)**			
Natural	191 (187–194.5)	185 (177.5–190)	**<0.0001**
Encoded	86 (75–96.5)	69 (46.5–83)	**<0.0001**
**EF Subdomain mOFC median (IQR)**			
**Stroop A**			
Errors	1 (0–3)	2 (1–3)	**0.0186**
Time	86 (70–110)	99 (77.5–120.5)	0.0591
Hits	81 (79–82)	80 (77–82)	0.1308
**Stroop B**			
Errors	1 (0–2)	2 (0.5–4)	**0.0102**
Time	82 (70–96)	91 (75–114.5)	**0.0252**
Hits	83 (82–84)	82 (80–83)	**0.0388**
**Card Game**			
Percentage of risk cards	35.1 (29.6–38.8)	37 (30.88–40.7)	0.2526
Total Score	28 (21–42)	25 (17–30.5)	**0.0446**
**Maze Test**			
Go through	0 (0–1)	0 (0–1)	0.1110
**Card Sorting**	0 (0–1.5)	1 (0–1)	0.2960
Maintenance errors			

Abbreviations: NW, normal weight; OW, overweight; OB, obesity; mOFC, Orbitomedial Cortex; IQR, interquartile range; BANFE-2, Neuropsychological Battery of Executive Functions and Frontal Lobes. A Mann–Whitney U test was used to compare differences between the normal-weight and overweight/obese groups. Statistically significant *p*-values are in bold.

**Table 4:** Executive function performance adolescents with normal weight and overweight/obesity according to gender.

	Female		Male	
BANFE-2	NW (*n* = 25)	OW/OB (*n* = 44)	NW (*n* = 16)	OW/OB (*n* = 13)
**mOFC, Total Score, median (IQR)**				
Natural	190 (184–195)	185 (177.3–190) **	192 (191–194.8)	183 (177.5–190) **
Encoded	83 (66–98)	69 (52.5–83) **	89 (86–97.25)	63 (46.5–83) **
**EF Subdomain mOFC median (IQR)**				
**Stroop A**				
Errors	1 (1–3)	2 (1–3)	1 (0–2.75)	3 (1–6.5) *
Time	89 (68.5–119)	99 (77.75–118.5)	83 (72.5–102.5)	97 (77.5–122.5)
Hits	81 (79–82)	81 (78–82)	80 (79–82.75)	77 (75–81.5) *
**Stroop B**				
Errors	1 (0–2)	2 (0.25–4) *	0.5 (0–2)	1 (0.5–3)
Time	84 (70–99.5)	90.5 (74–117)	73.5 (68.5–82.75)	94 (77.5–104) *
Hits	83 (82–84)	82 (80–83)	83 (82–84)	83 (80.5–83.5)
**Card Game**				
Percentage of risk cards	35.1 (30.2–39.75)	35.8 (33.08–40.7)	35.55 (29.6–38.8)	33.3 (27.78–40.72)
Total Score	28 (21–42)	23 (16.25–29) *	27.5 (21.75–40.5)	26 (18–41)
**Maze Test**				
Go through	0 (0–1)	1 (0–1)	0 (0–0)	0 (0–1)
**Card Sorting**				
Maintenance errors	0 (0–1)	1 (0–1)	1 (0–2)	1 (0–1)

Abbreviations: NW, normal weight; OW, overweight; OB, obesity; mOFC, Orbitomedial Cortex; IQR, interquartile range; BANFE-2, Neuropsychological Battery of Executive Functions and Frontal Lobes. * *p* < 0.05; ** *p* < 0.01. Mann–Whitney U test was used for comparison of differences in EF for gender.

**Table 5:** Association between the performance of EF and OW/OB in adolescents.

EF	OR	IC 95%	*p*-Value
Impaired	7.2485	2.9041 to 18.0918	<0.0001
Mild/Moderate Impairment	6.3	1.949 to 20.381	0.002
Severe Impairment	7.8	2.825 to 21.972	<0.0001

Abbreviations: OW, overweight; OB, obesity; EF, Executive Function. Reference group: normal weight.

## Data Availability

The original contributions presented in the study are included in the article; further inquiries can be directed to the corresponding author/s.
